# Directional sub-femtosecond charge transfer dynamics and the dimensionality of 1T-TaS_**2**_

**DOI:** 10.1038/s41598-018-36637-0

**Published:** 2019-01-24

**Authors:** Danilo Kühn, Moritz Müller, Florian Sorgenfrei, Erika Giangrisostomi, Raphael M. Jay, Ruslan Ovsyannikov, Nils Mårtensson, Daniel Sánchez-Portal, Alexander Föhlisch

**Affiliations:** 1 Institut für Physik und Astronomie, Universität Potsdam, Karl-Liebknecht-Str. 24/25, D-14476 Potsdam, Germany; 2 Helmholtz-Zentrum Berlin für Materialien und Energie GmbH, Albert-Einstein-Straße 15, D-12489 Berlin, Germany; 3 Centro de Física de Materiales CSIC-UPV/EHU and DIPC, Paseo Manuel de Lardizabal 5, E-20018 Donostia – San Sebastián, Spain; 4 CIC-nanoGUNE, Av. de Tolosa 76, E-20018 Donostia-San Sebastián, Spain; 5 Uppsala-Berlin joint Lab on Next Generation Electron Spectroscopy, Department of Physics and Astronomy, Uppsala University, Box 118, Uppsala, Sweden

## Abstract

For the layered transition metal dichalcogenide 1T-TaS_2_, we establish through a unique experimental approach and density functional theory, how ultrafast charge transfer in 1T-TaS_2_ takes on isotropic three-dimensional character or anisotropic two-dimensional character, depending on the commensurability of the charge density wave phases of 1T-TaS_2_. The X-ray spectroscopic core-hole-clock method prepares selectively in- and out-of-plane polarized sulfur 3p orbital occupation with respect to the 1T-TaS_2_ planes and monitors sub-femtosecond wave packet delocalization. Despite being a prototypical two-dimensional material, isotropic three-dimensional charge transfer is found in the commensurate charge density wave phase (CCDW), indicating strong coupling between layers. In contrast, anisotropic two-dimensional charge transfer occurs for the nearly commensurate phase (NCDW). In direct comparison, theory shows that interlayer interaction in the CCDW phase – not layer stacking variations – causes isotropic three-dimensional charge transfer. This is presumably a general mechanism for phase transitions and tailored properties of dichalcogenides with charge density waves.

## Introduction

Transition metal dichalcogenides (TMDs) are layered, quasi two-dimensional materials exhibiting phase transitions and tailored properties for sensors, electronic switching or energy storage^1–4^. In particular, 1T-TaS_2_ exhibits a complex phase diagram depending on temperature, doping or pressure encompassing charge density waves (CDW) with diverse commensurability and resistivity changes^5,6^. Interlayer CDW coupling and corresponding orbital textures are suggested to play a crucial role for these electronic properties^7–10^. For the nearly commensurate (NCDW) room temperature and the commensurate (CCDW) low temperature phases of 1T-TaS_2_, we determine the directional dependence of ultrafast charge transfer in the attosecond time domain with the core-hole-clock method^11,12^. The surprisingly strong interlayer coupling between the commensurate layers of the low temperature CCDW phase leads to isotropic three-dimensional charge transfer. In contrast, considerably slower out-of-plane over in-plane polarized charge transfer occurs for the nearly commensurate NCDW phase. This anisotropic two-dimensional charge transfer apparently reflects the reduced interlayer CDW coupling in the NCDW phase.

1T-TaS_2_, as a layered quasi-two-dimensional dichalcogenide, consists of repeated S-Ta-S slabs that are separated by a van der Waals gap. The phase diagram of 1T-TaS_2_ harbors several phases, which are characterized by periodic lattice distortions, accompanied by a charge density wave (CDW), and strong changes in resistivity. Below ≈180 K, 1T-TaS_2_ locks in a fully commensurate charge density wave (CCDW) phase with a strong periodic lattice distortion (up to 0.24 Å)^5,13^. A super lattice consisting of Star-of-David clusters builds up and the CDW wave vector is rotated by 13.9° with respect to the fundamental lattice. The CDW formation is mainly driven by a Peierls distortion of the lattice due to electron-phonon coupling. At room temperature, a nearly commensurate charge density wave (NCDW) forms having a wave vector rotated by 12° with respect to the fundamental lattice. Raising the temperature above ≈350 K, a fully incommensurate charge density wave (ICDW) phase appears.

Macroscopically, the NCDW-CCDW phase transition is accompanied by an abrupt increase of the electrical resistivity which has been assigned to the opening of a Mott-Hubbard gap at the Fermi energy^14^. Directional out-of-plane/in-plane ohmic resistivity anisotropy^5^ and switching to three-dimensional resistivity^4^ have been reported. The NCDW is commonly described as a structure of commensurate CDW domains separated by incommensurate domain walls which provide good electrical conductivity. The in-plane CDW structures are well investigated by, e.g., X-ray diffraction (XRD), electron diffraction and scanning tunneling microscopy (STM)^13,15,16^. However, the out-of-plane CDW stacking is still puzzling and periodicities and correlation lengths remain unclear.

Since the strong correlation between electronic and lattice subsystems cannot be decoupled in equilibrium, transient decoupling by ultrafast optical excitation and subsequent tracking of the relaxation dynamics has been carried out with photoelectron spectroscopy (PES), electron diffraction, or XRD^17–23^. These laser-based pump-probe experiments introduce a strong delocalized perturbation of the system with little directional selectivity (see Adhikary *et al*.^24^) and have difficulties to address the single femtosecond and sub-femtosecond timescales.

**Figure 1:**
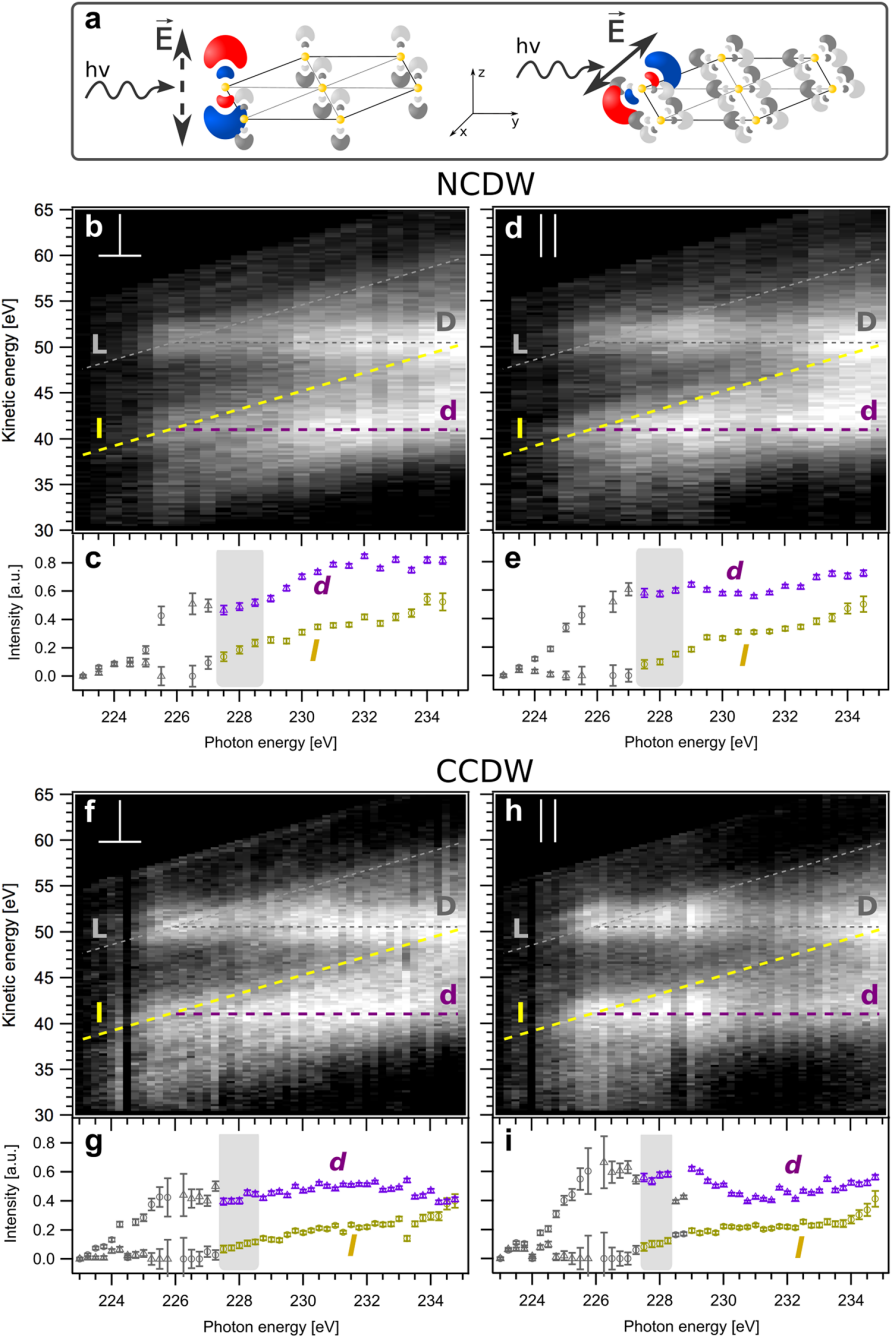
Core-hole-clock spectroscopy of in-plane and out-of-plane polarized sulfur 3p-excited states in the NCDW and CCDW phases of 1T-TaS_2_. (**a**) Selective electron excitation into the sulfur 3p_z_ (out-of-plane $\perp$) orbitals through the S2s → 3p_z_ dipole transition (left) and into the sulfur 3p_x,y_ (in-plane ||) orbitals through the S2s → 3p_x,y_ dipole transition (right) with linear polarized X-rays. (**b**,**d**,**f**,**h**) experimental sulfur L_1_L_23_M_123_ Coster-Kronig autoionization spectra of 1T-TaS_2_ for: **b** out-of-plane S2s → S3p_⊥_ excitation in the NCDW phase; (**d**) in-plane S2s → S3p_||_ excitation in the NCDW phase; **(f**) out-of-plane S2s → S3p_⊥_ excitation in the CCDW phase; (**h**) in-plane S2s → S3p_||_ excitation in the CCDW phase. Lighter colors depict higher intensity. Localized Auger-Raman decay channels for 2p^−1^3p^−1^3p^1^ (L) and 2p^−1^3s^−1^3p^1^ (l) final states show linear dispersion at constant binding energies. Auger decay channels for 2p^−1^3p^−1^deloc^1^ (D) and 2p^−1^3s^−1^deloc^1^(d) final states at constant kinetic energy open up at the resonance at hν = 226 eV. (**c**,**e**,**g**,**i**) intensities of the d- and l-channels are derived from fits (model described in the text) to the corresponding electron spectra in (**b,d,f** or **h**). Grey data points are excluded from quantitative intensity analysis due to spectral overlap. Shaded energy regions are used for quantitative charge transfer analysis.

To shed light onto the atomic origin of directional charge transfer and dimensionality, resonant soft X-ray spectroscopy is uniquely powerful, as it has been established for the directional attosecond charge transfer of adsorbed sulfur atoms on a Ruthenium (0001) surface^11,12^ and for charge carrier dynamics in a TMD^25^. In the core-hole-clock method an atomically localized electron wave packet is created, where the dipole selection rule in the absorption of linearly polarized soft X-rays leads to directional orbital population, i.e. the selective sulfur 3p_x_, 3p_y_, 3p_z_ occupation via the sulfur 2s → 3p transition. Figure 1a) depicts schematically how in-plane and out-of-plane sulfur 3p orbitals are selectively populated in this way with respect to the structural layers within the quasi-two-dimensional dichalcogenide 1T-TaS_2_. The excited state wave packet delocalization is then clocked against the atomic core-hole lifetime^11,26,27^, which is - as an elemental property - a well-defined quantity. Spectroscopically, the Auger resonant Raman (also called autoionization) channels of resonant photoelectron spectroscopy (RPES) show linear energy dispersion for localized states (no charge transfer) and constant kinetic energy features for the itinerant or delocalized states (charge transfer). Their intensity ratio is quantified and relates the charge-transfer time of the excited electronic state within the conduction bands to the core-hole lifetime. Thus, we can gain unique experimental insight into charge transfer as a function of commensurability and dimensionality of 1T-TaS_2_, based on the interplay of inter- and intra-layer charge density waves, van der Waals and covalent interactions, as well as the geometrical stacking contributions.

Figure 1 shows the polarization dependent RPES at the sulfur 2s → 3p transition of 1T-TaS_2_ in the room temperature NCDW phase (Fig. 1b out-of-plane, Fig. 1d in-plane) and in the low temperature CCDW phase at 30 K (Fig. 1f out-of-plane, Fig. 1h in-plane). In 1T-TaS_2_, the resonance of the S2s core-hole excitation occurs at hν = 226 eV independently of temperature and polarization. At 7° grazing X-ray incidence angle to the sample surface, p-polarized light excites S2s electrons predominantly into out-of-plane 3p_z_ orbitals (Fig. 1a left), while s-polarized light excites electrons predominantly into in-plane 3p_x,y_ orbitals (Fig. 1a right). All spectra are shown normalized to the incident photon flux and with a substraction of background from direct photoionization and multiple-electron-scattering (see SI Fig. 1).

Next to the linearly dispersing atomically localized 2p^−1^3s^−1^3p^1^ (l) and 2p^−1^3p^−1^3p^1^ (L) autoionization final states, delocalized charge transfer final states (or Auger channels) are found at constant kinetic energy (E_kin_ = 41.0 eV for the 2p^−1^3s^−1^deloc^1^ (d) and E_kin_ = 50.6 eV for the 2p^−1^3p^−1^deloc^1^ (D) final states, respectively). Below the resonance, the intensities of l,L increase with photon energy while insignificant intensities for the energetically forbidden d,D channels are observed. At resonance, d,D open up. All channels (l,L,d,D) are well-separated from each other and can be unambiguously quantified above the photon energy hν = 227,5 eV up to hν = 235 eV photon energy where the l- and D-channels cross. Although we analyze the full RPES range, we restrict the quantitative Raman fraction and charge transfer time analysis to the 227.5–228.5 eV photon energy range, just above the Fermi level. Here, the l- and d-channels are ideally separable and address the physically relevant density of states for charge transfer. As seen in Fig. 1, photon energies below lead to spectral overlap and ambiguity, as does the appearance of a second resonance between 229 eV and 230 eV. As pointed out in Föhlisch *et al*.^11^, only the l- and d-channels are used for the charge transfer analysis since the L- and D-channels interfere with the 3p^−1^ shake-up of the S2p main photoemission line. The intensities of the d- and l-features are fitted for all spectra with a model of a pair of spin orbit split (ΔE = 1.2 eV) Lorentzians at fixed width of Г = 3.3 eV FWHM. Peak positions are kept fixed at constant kinetic energy for the d-channel and at constant binding energy for the l-channel, respectively. Although approximate in line shape and detail, a global fit of all data with this model, based on the minimum physically motivated parameter set, describes all spectral features. Panels c,e,g,i of Fig. 1 show the polarization dependent intensities of the CCDW and NCDW phases from this fitting model and one particular slice of data at hν = 228 eV is presented as an example in Fig. 2 of the SI. We monitor the established splitting of the Ta4f photo emission line (see SI Fig. 3), as a direct measure of the CDW^28^, during the experiment. In the CCDW phase, long range coherence leads to a large Ta4f CDW splitting of ΔCDW = 0.62 eV. In contrast, the NCDW phase shows a splitting of only ΔNCDW = 0.48 eV.

**Figure 2:**
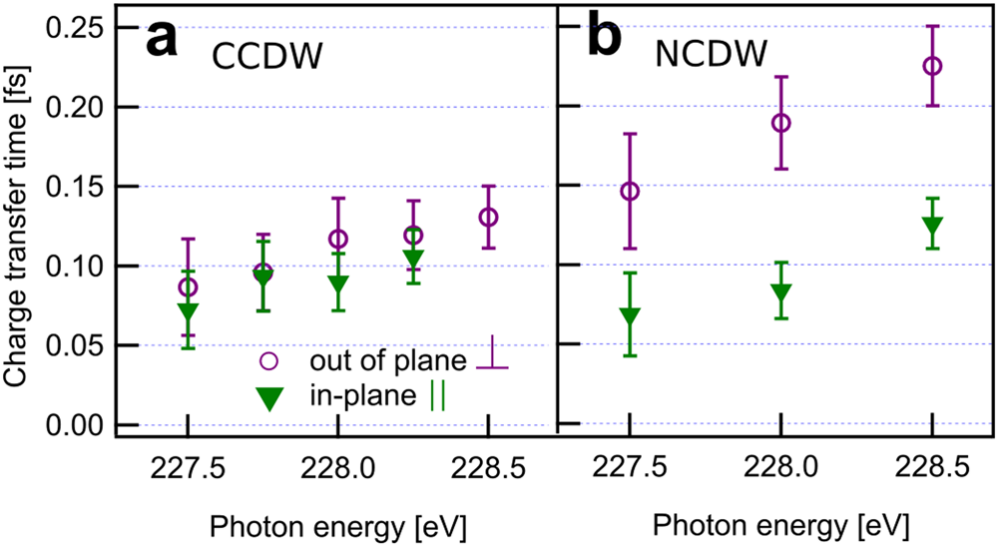
Quantitative charge transfer analysis of in-plane and out-of-plane polarized sulfur 3p-excited states in the NCDW and CCDW phases of 1T-TaS_2_. Charge transfer times in the CCDW low temperature phase (**a**) and in the NCDW room temperature phase (**b**) for in-plane S2s → 3p_||_ (green triangles) and out-of-plane S2s → 3p_⊥_ (purple circles) excitation as determined from the Auger-Raman fraction of the L_1_L_2/3_M_1/2/3_ (l,d) decay in the shaded photon energy ranges in Fig. 1. Error bars are derived from standard deviations of the fits.

We can now convert these l- and d-intensities to charge transfer times *τ*_*ct*_ via *τ*_*ct*_/*τ* = l/d for all combinations of NCDW and CCDW phases and in-plane and out-of-plane orbital polarizations, respectively^11^. In this model of single exponential decay and with the S2s core-hole lifetime of *τ = *0.5 fs, we summarize the phase and orbital polarization dependent charge transfer times in Fig. 2. In the CCDW phase (see Fig. 2a), no variation of charge transfer times is observed for different orbital polarizations in the conduction band states excited between 227.5 and 228.5 eV photon energy. In contrast, the NCDW phase (see Fig. 2b) shows a strong anisotropy of the charge transfer times. In particular, the NCDW in-plane polarized charge transfer time is equal to the CCDW charge transfer time, but the out-of-plane charge transfer of NCDW is two times slower than for in-plane excitation.

These experimental findings point strongly towards the following scenario: Despite the layered nature of all phases of 1T-TaS_2_, a strong three-dimensional character of the CCDW phase prevails, which is reduced to a more two-dimensional character with the loss of commensurability towards the NCDW phase. Our observations are thus resonating with some previous findings: The build-up of domains in the NCDW phase at reduced long range in-plane coherence of the CDW^29^. A recent transmission electron microscopy experiment with high spatial resolution indicates a partial increase of CDW ordering along the c-axis during the NCDW to CCDW phase transition^30^. Experiments with nano-structured devices^7^ or synthesized few-layer 1T-TaS_2_^31^ observe a CDW collapse at sample thicknesses below 10 nm and a recent ARPES experiment postulates a metallic band perpendicular to the crystal layers^10^ that forms in the CCDW phase. All these findings go in line with our observation that the two-dimensional character of the CDW is weakened going from the NCDW phase to the CCDW phase, where at least electronically a three-dimensional character sets in for the CCDW phase of 1T-TaS_2_, that seems to be intrinsically linked to the formation and stabilization of the CCDW phase. Also, stacking in the CDW phase has been shown to critically affect the dispersion of bands crossing the Fermi level and can lead to an opening of gaps in the in-plane direction^9^.

**Figure 3:**
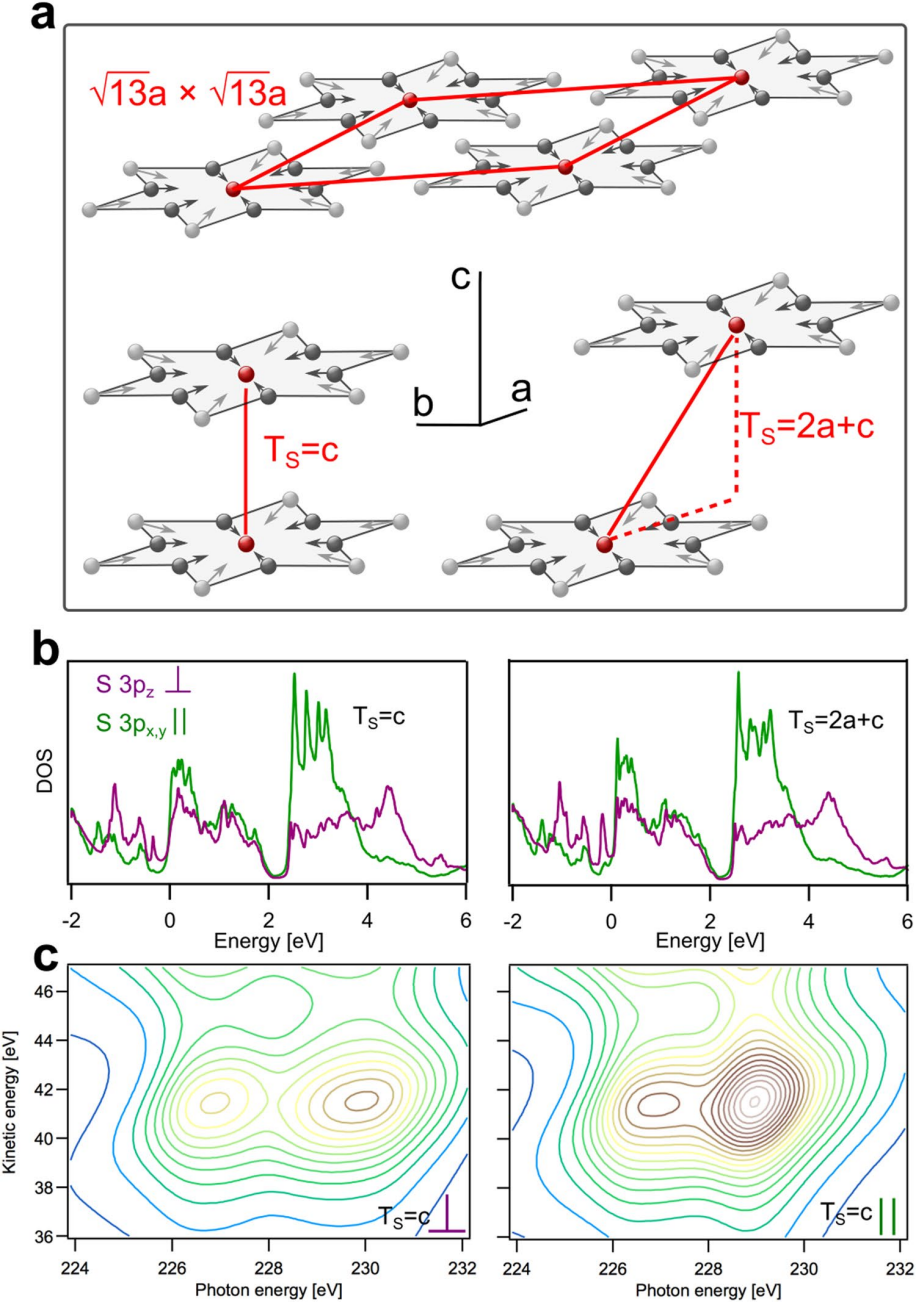
DFT calculations of electronic structure and spectral signatures of 1T-TaS_2_ projected on S3p orbitals for two commensurate CDW interlayer stacking situations. (**a**) illustration of the $\sqrt{13}a\times \sqrt{13}a$ “Star-of-David” reconstruction of the Ta layers caused by the charge-density wave/periodic latice distortion (CDW/PLD) in the NCDW and CCDW phases. All atoms of each star are displaced (depicted with arrows) towards the center atom. In the CDW structure, three different charge densities at the Ta sites and five inequivalent S sites exist. Red arrows span the reconstructed elementary cell which contains 13 Ta atoms. Two possible interlayer stackings are depicted (bottom). Left: CDW stacking of the Ta planes with the Star-of-David centers on top of each other (T_s_ = c). Right: CDW stacking with a stacking vector having an in-plane component (T_s_ = 2a + c). (**b**) DFT calculations of the partial DOS of the polarized S3p states with on-site S2s core hole for T_s_ = c (left) and T_s_ = 2a + c (right). Purple shows the out-of-plane S3p_⊥_ PDOS and green shows the in-plane S3p_||_ PDOS. The Fermi level is located at 0 eV. (**c**) simulations of the sulfur L_1_L_23_M_123_ autoionization scattering planes based on T_s_ = c partial DOS from (**b**) for S2s → 3p_⊥_ transition (left) and S2s → 3p_||_ transition (right). Intensity increases from blue to brown color.

To identify the physical drivers further, we investigate computationally the influence of stacking variations on charge transfer times in the CCDW phase. With density functional theory (DFT), we have calculated the electronic structure and the S2s core-excited 1T-TaS_2_ electronic density of states (DOS) in the CCDW phase of 1T-TaS_2_ for the two well-chosen perpendicular stacking situations T_s_ = c and T_s_ = 2a + c (see Fig. 3a), extracting in a next step the influence of stacking on the anisotropy of charge transfer. Both CDW stackings and combinations of them are likely to occur in the NCDW and CCDW phases according to various XRD measurements^9,32,33^, transmission electron diffraction^34^ and calculations^35,36^.

Figure 3a illustrates the hexagonal crystal structure of the Ta layers in the CCDW phase. Within a layer, the commensurate cluster super structure is depicted consisting of 13 Ta atoms per star. In the CDW structure three different charge densities at the Ta sites and five inequivalent S sites exist. Between the layers, different stacking situations of the Star of David clusters are possible. The two depicted T_s_ = c and T_s_ = 2a + c stacking situations are, with regard to their Coulomb interaction between adjacent layers, the extremal arrangements for CDW stacking in 1T-TaS_2_. The T_s_ = c stacking is energetically unfavorable due to S-S Coulomb repulsion. The T_s_ = c stacking pairs the strongly out-of-plane displaced S atoms at the Star-of-David centers in adjacent layers^15,29^. In contrast, the T_s_ = 2a + c stacking pairs the strongly displaced sulfur atom in the Star-of-David center with a weakly displaced sulfur atom between the Star-of-David centers.

Figure 3b displays the DOS projected on the S3p_⊥_ and S3p_||_ states for these T_s_ = c and T_s_ = 2a + c stacking situations. For both stackings the projected DOS (PDOS) is distributed over a wide energy range, reflecting the covalent bonding with neighboring atoms in the material. The DOS appears split around 2.2 eV above the Fermi level in accordance with the observations made in inverse photoemission experiments^37,38^. Also, all autoionization spectral features for out-of-plane and in-plane excitation can be modeled based on the PDOS as shown in Fig. 3c and show good agreement with experiment. Keep in mind that the convolution of the PDOS yielding autoionization maps, as described in the Supporting Information, neglects channel interference and over-represents the localized autoionization channels. However, the overall good agreement of the simulations enables to assign our experimental charge transfer analysis to the first spectral feature observed in the calculations between E_Fermi_ and the bandgap at about 2.2 eV. As a result, DFT indicates, that despite T_s_ = c and T_s_ = 2a + c should give significant CDW stacking related deviations, remarkable similarity of the PDOS for T_s_ = c and T_s_ = 2a + c prevails.

To finally analyze the elastic lifetimes of the computed S3p-resonances, we transform the theoretical results to the time-domain. We make an estimate of the elastic lifetime considering the time evolution of a resonance living on the finite energy interval between the Fermi level and the band gap 2.2 eV above (see methods section for details). The calculated elastic lifetimes of the core-excited S3p resonances are, for the T_s_ = c stacking, τ_(S3px)_ = 1.21 ± 0.15 fs, τ_(S3py)_ = 1.19 ± 0.15 fs and τ_(S3pz)_ = 1.19 ± 0.06 fs and, for the T_s_ = 2a + c stacking, τ_(S3px)_ = 1.20 ± 0.15 fs, τ_(S3py)_ = 1.19 ± 0.14 fs and τ_(S3pz)_ = 1.18 ± 0.08 fs. The numbers are given as weighted averages over the chemically inequivalent sulfur sites with error bars as standard deviations. Since the strongly hybridized character of the sulfur atoms leads to structured and broad S3p-projected spectra from the present set of calculations, a Lorentzian line shape fit to determine the lifetime from its resonance width is unsuitable. Thus, in the present case of 1T-TaS_2_ a similar time-domain procedure has been adopted as previously for the broad resonances encountered in the case of chemisorbed sulfur on ruthenium^11^, extending the Lorentzian line shape fit applied to weak coupling scenarios such as physisorbed atoms on surfaces^39,40^. The result of our theoretical model shows negligible dependence of the charge transfer time on the symmetry of the initial wave packet and no dependence on interlayer stacking. DFT, as a 0 K theory, is thus qualitatively in full agreement with the low temperature CCDW phase experiment, although the absolute numbers of charge transfer differ somewhat on the 1.2 fs vs 0.2 fs timescales, respectively. Thus, our observations strongly support that interlayer coupling in the CCDW phase creates a three-dimensional character of the electronic structure at low temperatures. In contrast, the room temperature NCDW phase is characterized by the unlocking of interlayer coupling, where the gradual loss of commensurability brings the two-dimensional character of the layers within 1T-TaS_2_ to the forefront, as is experimentally seen by the two times slower out-of-plane charge transfer.

## Summary

1T-TaS_2_ exhibits, despite of being a prototypical layered dichalcogenide, surprisingly strong electronic interlayer coupling between the commensurate layers of the low temperature CCDW phase, leading to isotropic three-dimensional charge transfer on the sub-femtosecond timescale. In contrast, two times slower out-of-plane than in-plane polarized charge transfer occurs for the nearly commensurate NCDW phase. This reflects the apparently reduced interlayer CDW coupling in the NCDW phase as the loss of commensurability sets in, resulting in anisotropic two-dimensional charge transfer. Uniquely selective preparation of the propagating excited state wave packet and sub-femtosecond temporal information from the soft X-ray spectroscopic core-hole-clock method allows to establish, in direct comparison to density functional theory, this presumably general mechanism for phase transitions and tailored properties of layered dichalcogenide materials involving interlayer coupling and charge density wave physics.

## Methods

### XPS

All experimental data were measured at the BESSY II UE56/1 PGM soft X-Ray beamline in the single bunch mode with 13 mA ring current. The photon energy resolution is 110 meV, the mean photon flux 10^8^ ph/s at hν = 230 eV and the sample spot size 200 × 200 µm^2^. The electron analyzer is an ARTOF-2 angle-resolved time of flight spectrometer by Scienta-Omicron with 56° full cone angular acceptance. The estimated kinetic energy resolution is better than 20 meV in the whole energy range under consideration. The sample surface is vertically oriented. The photon beam impinges under 7° on the sample surface. The spectrometer is in the same plane as the photon beam, with the optical axis at 50° with respect to it. Samples were cooled with a Janis ST-400 cryostat down to 30 K. The base pressure was below 2∙10^−10^ mbar. All samples were cleaved under vacuum conditions at low temperature. Sample quality was checked by measuring the CDW splitting of the Ta4f XPS lines.

### Sample production

1T-TaS_2_ single crystals were grown using iodine vapor transport with excess sulfur (2.5 mg/cm^3^) in a temperature gradient of 840–870 C for 1000 h^18^.

### Data analysis

Raw spectra were normalized against the storage ring current to compensate for photon intensity variations due to the synchrotron Top-Up operation mode. To extract the relevant autoionization decay channels, all resonant PES maps were individually analyzed in the following way:

First of all, an exponential background of the form ${I}_{B}({E}_{kin})={a}_{h\nu }\cdot [1-b\cdot \exp (-\alpha \cdot {E}_{kin})]$, which describes the low energy secondary electron cascade and the high energy XPS loss tail, was subtracted from all electron spectra. The parameters $b=13.5$ and $\alpha =0.07$ were kept constant for all spectra of all maps, assuming that the electron cascade energy distribution is only weakly influenced by sample temperature and X-ray polarization. The amplitude parameter ${a}_{h\nu }$ was determined by fitting *I*_*B*_ to the spectrum at hν = 223 eV in the kinetic energy range between 63 and 64.5 eV, which is well above the S2p XPS lines, and then scaled for all other photon energies by using the measured total electron emission current as the scaling factor. An exception is the CCDW in-plane polarization map, where the electron emission current was offset. In the latter case, we have instead used an averaged ${a}_{h\nu }$ calculated from the other three settings. In a next step, we extracted the S2p direct photoemission spectrum, including the main lines [E_B_(S2p_1/2_) = 162.4 eV; E_B_(S2p_3/2_) = 161.2 eV] and the corresponding shake-up, from the electron spectrum at hν = 223 eV (far below the S2s resonance) and subtracted it from the electron spectra at constant binding energy for each photon energy, by scaling the combined S2p XPS and shake-up intensity with the S2p_1/2_ intensity.

This gives the RPES maps as shown in Fig. 1.

In a last step the intensities of the localized (l,L) and delocalized (d,D) channels were determined by applying a fit with equal line shape to each feature. Each peak consists of two Lorentzians with 3.3 eV FWHM, a spin orbit splitting of 1.2 eV and an intensity ratio of 1:2. The peak positions were fixed at constant kinetic energy for d, D and constant binding energy for l, L and are identical at the resonance (hν = 226 eV).

Figures which illustrate the data analysis procedure can be found in the supplementary material.

### DFT calculations

Density Functional Theory (DFT) as implemented in the Siesta code^41^ is used to compute the electronic structure of 1T-TaS_2_ in the CCDW phase. The calculations employed the vdW-DF by Dion *et al*.^42^ with the optimized exchange by Klimes *et al*.^43^. While this level of theory is not expected to reproduce all the details of the band structure close to the Fermi level, such as the correct sizes of experimentally observed pseudogaps, a fairly good overall agreement with spectroscopic results has been reported^9^.

The atom centered basis set included double-ξ and polarization orbitals^44^ with an energy-shift of 0.1 eV for the confinement of the orbitals. The bulk material showing the David star reconstruction and a commensurate CDW below ≈180 K was simulated by setting-up the known $\sqrt{13}a\times \sqrt{13}a$ hexagonal super cell containing 13 Ta atoms^13^.

Subsequent geometry relaxation produced a displacement of the surrounding Ta atoms towards a central one and outward buckling of the sulfurs around the center of contraction.

The forces were relaxed with a threshold of 25 meV/Å. We used a 5 × 5 × 9 Monkhorst-Pack k-point sampling, a mesh cutoff of 200 Ry, and an electronic temperature of 300 K.

The lattice constants a = 3.398 Å and c = 5.910 Å of the undistorted material’s unit cell (containing a single unit of TaS_2_) were taken from relaxation runs with a stress tolerance of 0.1 GPa and a k-point sampling of 20 × 20 × 10. The lattice constants are in good agreement with the ones determined experimentally^29^. The final slab calculations with a fixed cell employed 9 layers and the outer 2 layers on each side have been relaxed.

The surface Brillouin zone was sampled with 5 × 5 k-points and 30 Å of vacuum were added. We used a hexagonal super cell to simulate the T_s _= c CDW stacking^15,29^ and a triclinic super cell for the T_s_ = 2a + c stacking, respectively.

To simulate core-excited sulfur atoms in line with the presented spectroscopies, we used pseudopotentials generated from an electronic configuration with a hole in the 2s-state.

The positive charge of the hole was subsequently balanced by an additional electron in the valence. Such core-excited sulfur atoms were placed symmetrically on both sides of the slab in a dipole canceling set-up. We accounted separately for five possible excitations at inequivalent sites in the outermost sulfur layer of the material. We assumed that the initial excitation produces an intermediate state, where a S2s-electron has been promoted into a S3p-state following atomic dipole selection rules. This intermediate resonance wave packet was modeled by a linear combination of S3p-orbitals

$$|{{\rm{\Phi }}}_{R}(\theta ,\varphi )\rangle =\,\sin \,\theta \,\cos \,\varphi |{p}_{x}\rangle +\,\sin \,\theta \,\sin \,\varphi |{p}_{y}\rangle +\,\cos \,\theta |{p}_{z}\rangle $$

with the angles *ϕ* and *θ* reflecting the polarization vector of the incident light.

The spectra were then calculated by projecting the spectral density onto the resonance wave packet ${\rho }_{R}(E)=-\,\frac{1}{\pi }\Im \langle {\varphi }_{R}|\hat{G}(E)|{\varphi }_{R}\rangle =-\,\frac{1}{\pi }[{G}_{RR}(E)]$ using a Green’s function approach^39,40^.

We implemented the required projection scheme into the TBTrans tool of the Siesta/TranSiesta package^41,45,46^. The tool effectively accounts for a semi-infinite substrate.

After defining the out-of-plane direction $|{p}_{z}\rangle$, we computed the mean in-plane spectrum from the expression:

$$\frac{1}{2\pi }{\int }_{0}^{2\pi }d\varphi \langle {\varphi }_{R}(\frac{\pi }{2},\varphi )|\hat{G}(e)|{\varphi }_{R}(\frac{\pi }{2},\varphi )\rangle =\frac{1}{2}\langle {p}_{x}|\hat{G}(E)|{p}_{x}\rangle +\frac{1}{2}\langle {p}_{y}|\hat{G}(E)|{p}_{y}\rangle $$

We obtained the final spectra in a weighted average over the excitations at the five distinct sulfur sites.

To extract estimates of the elastic lifetimes from the projected density of states ${\rho }_{R}(E)$ in the energy range from the Fermi energy to the electronic gap at ~2.2 eV above, the real part of the projected Green’s function was reconstructed using the Hilbert transform. Subsequent Fourier transformation of the Green’s function yields the survival amplitude $A(t)=\langle {\varphi }_{R}(t=0)|{\varphi }_{R}(t)\rangle$ of the initial wave packet, since $\tilde{A}(t)=\frac{i}{\pi }{G}_{RR}(E)$. The value for the lifetime of the resonance at one specific sulfur site was then defined as the smallest time *t* at which ${|A(t)|}^{2}\le {e}^{-1}$. When doing this, care must be taken to subtract the effect due to the small imaginary part added to the energy, when computing the Green’s function using recursive methods (20 meV in the present case).

## Electronic supplementary material

Related Manuscript File
