# Supplementary material for: Directional sub-femtosecond charge transfer dynamics and the dimensionality of 1T-TaS2
Source: Sci Rep. 2019 Jan 24;9:488. doi: 10.1038/s41598-018-36637-0 (PMC6346016; doi:10.1038/s41598-018-36637-0)
Supplement: Supplementary file 1 — Related Manuscript File [file 41598_2018_36637_MOESM1_ESM.pdf]

Supplementary material:

Directional sub-femtosecond charge transfer dynamics and the dimensionality of 1T-TaS_2

Authors:
Danilo Kühn[1,2], Moritz Müller[3,4], Florian Sorgenfrei[1,2], Erika Giangrisostomi[2], Raphael M. Jay[1], Ruslan Ovsyannikov[2], Nils Mårtensson[5], Daniel Sanchez-Portal[3] and Alexander Föhlisch[1,2,*]

Affiliations:

[1]Institut für Physik und Astronomie, Universität Potsdam, Karl-Liebknecht-Str. 24/25, D-14476 Potsdam, Germany

[2]Helmholtz-Zentrum Berlin für Materialien und Energie GmbH, Albert-Einstein-Straße 15, D-12489 Berlin, Germany

[3]Centro de Física de Materiales CSIC-UPV/EHU and DIPC, Paseo Manuel de Lardizabal 5, E-20018 Donostia – San Sebastián, Spain

[4]CIC-nanoGUNE, Av. de Tolosa 76, E-20018 Donostia-San Sebastián, Spain

[5]Uppsala-Berlin joint Lab on Next Generation Electron Spectroscopy, Department of Physics and Astronomy, Uppsala University, Box 118, Uppsala, Sweden

* E-mail: alexander.foehlisch@helmholtz-berlin.de

RPES calculations:

The resonant photoemission spectra in Fig. 3c were calculated with the polarized S3p ρ^{3p} and the S3s ρ^{3s} core-excited PDOS by using the following modified formulas from Drube et al.[1] and Föhlisch et al.[2] taking a possible S 3s and S 3p valence hybridization and the S 2p spin orbit splitting $\Delta E_{SO} = 1.2$ eV into account:

$$I(E_A, h\nu) = C \int_{E_F}^{\infty} dE \int_{-\infty}^{E_F} dE' \frac{\rho^{3p}(E)}{(h\nu - E - E_{S2s})^2 + \frac{\Gamma_{S2s}^2}{4}} \cdot \frac{\rho_{SO}^{3s/3p}(E')}{\left(h\nu - E - E_A - E_{S2p_{3/2}} - E'\right)^2 + \frac{\Gamma_{S3s,S3p}^2}{4}} \quad (2)$$

$$\rho_{SO}^{3s/3p}(E') = \int d\epsilon \, [\rho^{3s}(E') + \rho^{3p}(E')][\delta(\epsilon - E') + 2\delta(\epsilon - E' + \Delta E_{SO})] \quad (3)$$

Here $h\nu$ is the X-ray photon energy, E_A the kinetic energy of the autoionization decay channel, $E_{S2p1/2}$ =162.4 eV and $E_{S2p3/2}$ =161.2 eV the experimentally determined binding energies of the S 2p doublet state and E_{S2s} =226 eV the binding energy of the S2s state. $\Gamma_{S2s} = 1.5$ eV is the life time width from the S 2s core hole, $\Gamma_{S3s,S3p} = 3.3$ eV is the same phenomenological width as in the fits and C is a constant prefactor, which takes the matrix elements into account. The simulated scattering planes are shifted about 2 eV to lower kinetic energy to match the experimental data. The discrepancy can be explained by the

fact that the $2p^{-1}3s^{-1}$ two hole final state has a higher binding energy than the sum of individual binding energies due to Coulomb interaction.

Supplementary Figures:

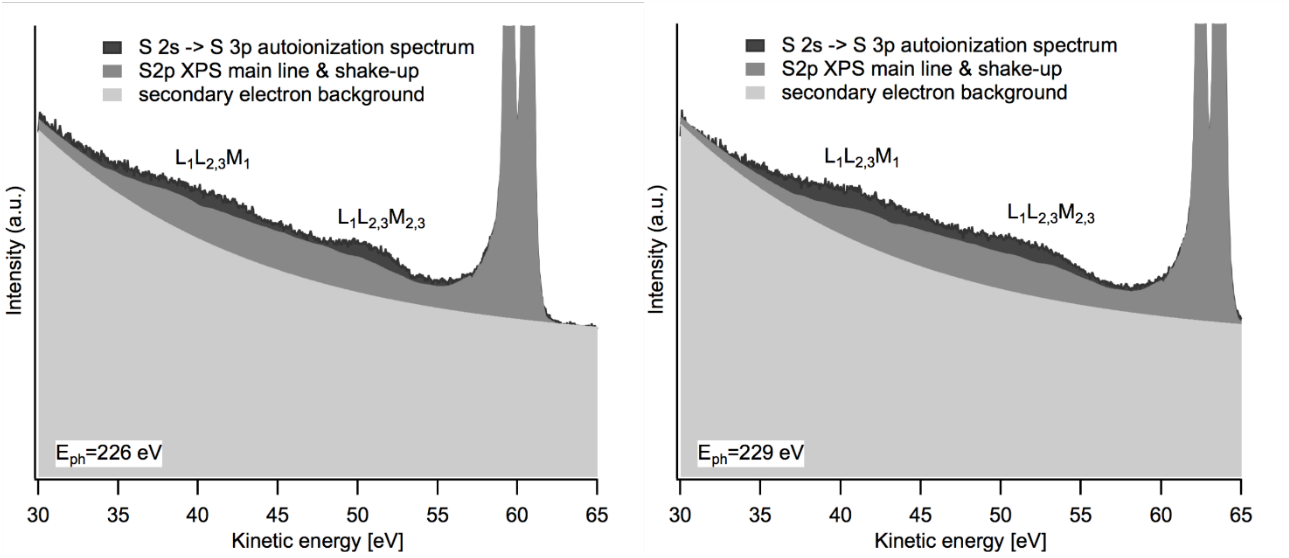

Figure 1: Illustration of the spectral decomposition for two different X-Ray excitation energies.

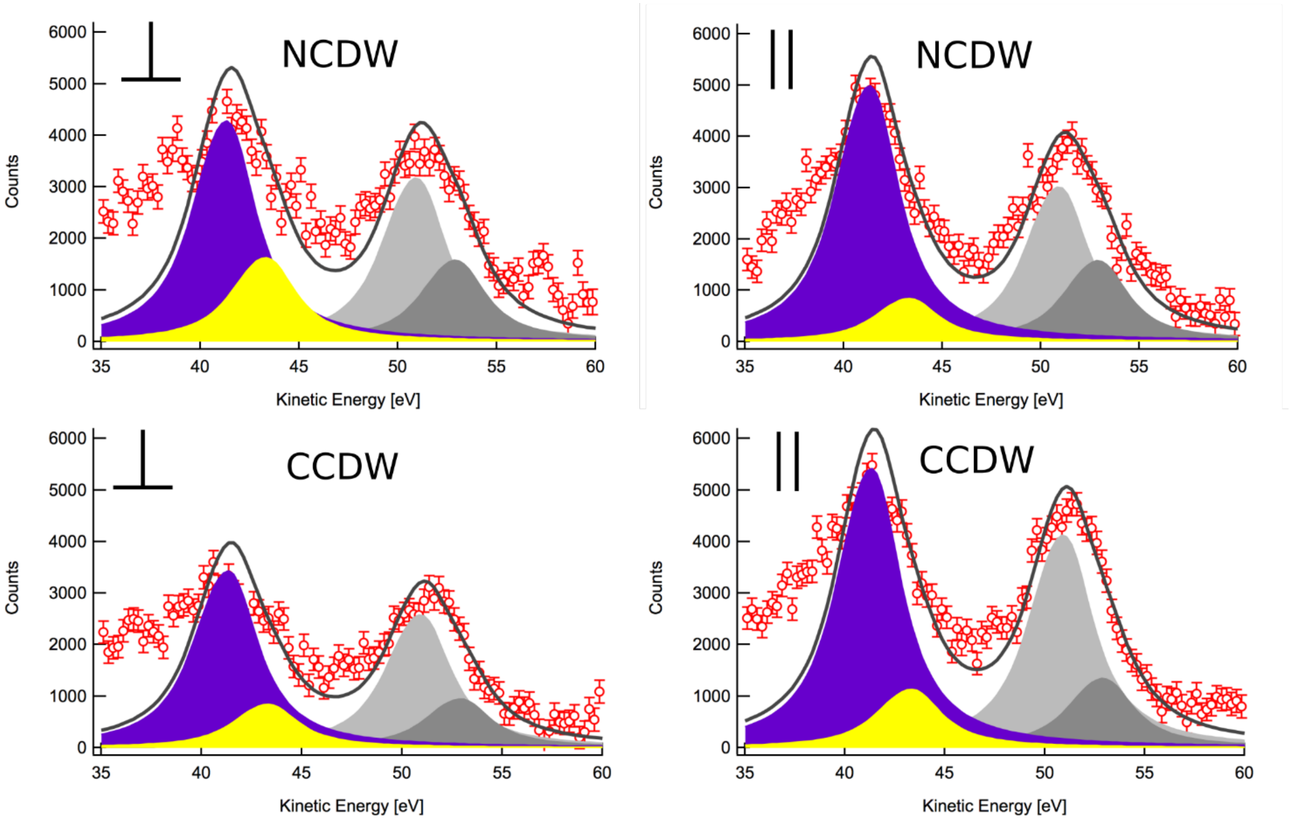

Figure 2: Illustration of the fit to the autoionization spectra at hv=228 eV. The four spectral components d (purple), l (yellow), D (light grey), L (dark grey) are shaded. The black line is the sum of them and matches the experimental data within the region of interest reasonably.

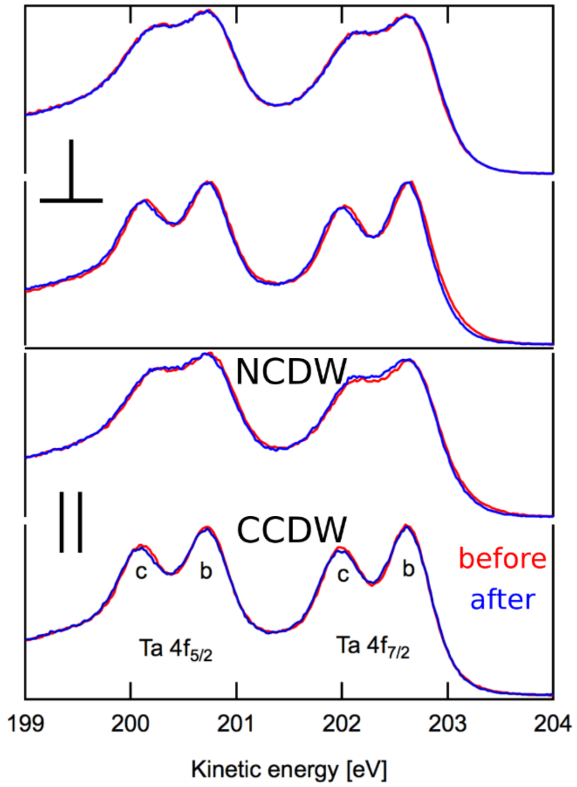

Figure 3: Ta 4f XPS spectra attesting sample quality
Ta 4f XPS spectra can be used to monitor the CDW/PLD amplitude and are therefore an indicator for sample quality. The splitting of the two chemically inequivalent Ta sites (*b* corresponds to the 6 inner atoms of a Star of David cluster and *c* corresponds to the 6 outer atoms of a Star of David cluster) is directly proportional to the CDW amplitude. No significant differences can be observed between spectra taken before and after recording resonant PES maps.

References:

1. Drube, W.; Treusch, R. & Materlik, G. Density of State Effects in Ag $L_3M_{4,5}M_{4,5}$ Threshold Auger Spectra *Phys. Rev. Lett., American Physical Society,* **1995***, 74*, 42-45

2. Föhlisch, A.; Karis, O.; Weinelt, M.; Hasselström, J.; Nilsson, A. & Mårtensson, N. Auger Resonant Raman Scattering in Itinerant Electron Systems: Continuum Excitation in Cu *Phys. Rev. Lett., American Physical Society,* **2001***, 88*, 027601
